# Domestic Animals vs. Man

**Published:** 1891-12

**Authors:** 


### DOMESTIC ANIMALS VS. MAN.

Mr. Harrison, in his message to Congress, in speaking of the work of the Department of Agriculture, says.

"If the establishment of the Department of Agriculture is regarded by any one as a mere concession to the demand of a worthy class of people, that impression has been most effectually removed by the great results already attained. Its home influeace has been very great in disseminating agricultural and horticultural information, stimulating and directing a further diversification of crops, in detecting and eradicating diseases of domestic animals, and more than all, in the close and informal contact which it has established and maintains with the farmers and stock raisers of the whole country. Every request for information has had prompt attention, and every suggestion merited considera. tion. The scientific corps is of high order, and is pushing its investigations with method and enthusiasm."

That is all right, and as it should be. We only wish to remark that no "concession," even, has ever been made to the demands of a certain other "worthy class," in a matter which some of us consider of much more importance than the health of cattle or "domestic animals." The American Medical Association is now petitioning Congress, or will shortly memorialize them, to create a department of PUBLIC HEALTH, through which there may be disseminated sanitary information; and diseases which destroy the *human family* may be "detected and eradicated." Many efforts have been made by leading physicians through influential Congressmen to have some such step taken; to appoint a Minister of Public Health, or Secretary of Public Health or something whereby the same care of the public health and supervision of public sanitation may be instituted and exercised, as obtains in the case of domestic animals; but without avail. It appears difficult, if not impossible, to convince legislators that medical men should be consulted, at least, in the enactment of sanitary laws; and as the case now stands, not only with regard to Congress but with our legislature, more thought is given to the protection of the dumb brutes than to the safety of the people. It is a paradox. Should Congress create a Department of Public Health, as it should, by every consideration, do, "every request for informa tion" on *sanitary* subjects would also receive prompt attention; and the President in his next annual address could say, "its influence has been very great in detecting and eradicating" contagious and infectious diseases which carry off annually thousands of people.

In the name of justice, reason and common sense, let us have a Department of Public Health. Is man not worthy of as much consideration as his horse, or his sheep or cattle?

